# Temporal trends of the use of dexamethasone for the treatment of bronchopulmonary dysplasia in very low-birth-weight preterm infants: a single-center evaluation

**DOI:** 10.31744/einstein_journal/2024AO0849

**Published:** 2024-10-23

**Authors:** Mei Li Ng Teixeira, Sérgio Tadeu Martins Marba, Jamil Pedro de Siqueira Caldas

**Affiliations:** 1 Universidade Estadual de Campinas Faculdade de Ciências Médicas Campinas SP Brazil Faculdade de Ciências Médicas, Universidade Estadual de Campinas, Campinas, SP, Brazil.; 2 Universidade Estadual de Campinas Department of Pediatrics Campinas SP Brazil Department of Pediatrics, Universidade Estadual de Campinas, Campinas, SP, Brazil.

**Keywords:** Infant, very low birth weight, Infant, premature, Dexamethasone, Bronchopulmonary dysplasia, Trends

## Abstract

**Objective:**

To evaluate temporal trends in the use of dexamethasone for the treatment of bronchopulmonary dysplasia in very-low-birth-weight preterm infants.

**Methods:**

A cohort study was conducted using an electronic database and medical records of all very low-birth-weight preterm infants admitted to a university tertiary neonatal unit between 2006 to 2022. The main outcome was the use of systemic dexamethasone to treat bronchopulmonary dysplasia, regardless of the dose or duration of treatment. Annual rates were evaluated using the Cochran-Armitage test.

**Results:**

A total of 1,691 very-low-birthweight preterm infants were admitted during the study period, with a median birth weight of 1100 g (interquartile range [IQR] 850-1300g) and a median gestational age of 29 weeks (IQR= 27-31g). Infants exposed to dexamethasone were smaller (birth weight: 765 *versus* 1134g, p<0.001) and more immature (gestational age: 28 *versus* 31 weeks, p<0.001). The overall rate of dexamethasone use was 9.6% (annual variation 6.7% to 13.9%) and remained stable over the study period (p=0.287), including in infants aged <32 weeks (12.0%; p=0.203) and <28 weeks (24.6%; p=0.851). Mechanical ventilation and mortality rates remained stable at 58.8% (p=0.435) and 14.5% (p=0.078), respectively. However, the birth rate at <28 weeks of gestation increased (28.8%, annual variation of 16.0% to 43.8%, p<0.001).

**Conclusion:**

Approximately one in 10 preterm very low-birth-weight infants and one in four of those <28 weeks received dexamethasone, with a trend towards stable use over time, despite a significant trend towards an increase in extreme preterm newborn infants.

## In Brief

In this large single-center evaluation, approximately one in 10 preterm very low-birth-weight infants and one in four of those <28 weeks were exposed to postnatal dexamethasone due to bronchopulmonary dysplasia. There was a trend towards stability of the use rate over time, despite a significant trend towards an increase in extreme preterm newborn infants.

## Highlights

■ Mechanical ventilation and mortality rates remained stable.
■ The birth rate at <28 weeks' gestation increased during the evaluation period.
■ The overall rate of dexamethasone administration was low; however, its use was more significant in infants aged <28 weeks.

## Graphical abstract

**Figure:**
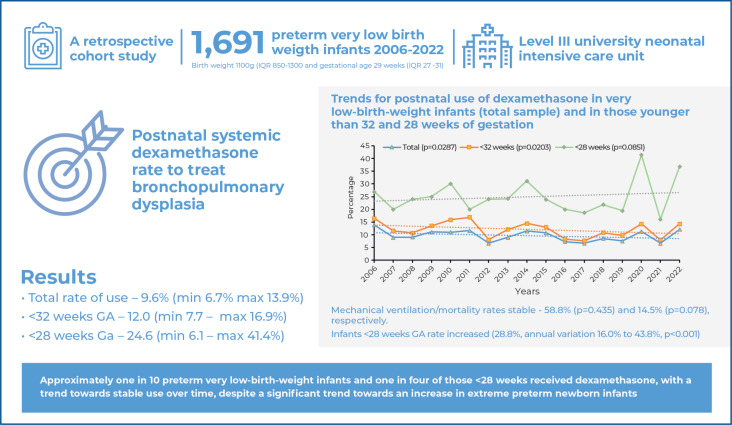

## INTRODUCTION

Despite advances in neonatal care over the last few decades, bronchopulmonary dysplasia (BPD) remains a relevant clinical and public health problem. It constitutes one of the main causes of chronic respiratory diseases in childhood, leading to repeated and prolonged hospitalization associated with high mortality rates and changes in growth and neurodevelopment.^(1)^

The incidence of BPD is inversely proportional to the gestational age and birth weight. In two contemporary evaluations of extremely preterm American infants, the rates of oxygen dependence at 36 weeks postmenstrual age (PMA) ranged from 44.8% during 2008-2012 ^(2)^ to 49.8% more recently (2013-2018).^(3)^ In both analyses, the rates of the disease at 23 and 24 weeks of gestation were more than double those in infants born at 28 weeks of gestation. An evaluation by the Brazilian Neonatal Research Network (RBPN - *Rede* *Brasileira de Pesquisa Neonatal*) showed an incidence of 20% in preterm very low-birth-weight infants (VLBW) who survived hospitalization^(4)^ which was similar to the findings of Lee et al. in California.^(5)^

In the 1990s, corticosteroids were widely administered postnatally to reduce the incidence of BPD. Prolonged courses of high-dose corticosteroids have become increasingly common. However, an increase in short- and long-term complications has been observed, including hyperglycemia, arterial hypertension, an increased risk of infections, adrenal suppression, and reduced growth. In addition, its use is associated with an increased incidence of cerebral palsy and neurodevelopmental delays.^(6,7)^ Therefore, in 2002, the American Academy of Pediatrics and the Canadian Society of Pediatrics formally discouraged the use of corticosteroids, especially at high doses, in the context of BPD because of their association with cerebral palsy.^(8)^

Publications restricting the use of dexamethasone have significantly reduced its use in high-income countries. However, recent data from the Vermont Network revealed a trend towards a gradual increase in its use in newborns born at <29 weeks of gestation, with a rate of use close to 20%, which is twice that observed in the early 2000s.^(9)^ This has been verified in studies in other developed countries such as England, Wales^(10)^ and France.^(11)^ Moreover, a database analysis grouping seven neonatal research networks from high-income countries that participated in the International Network for Evaluating Outcomes of Neonates (iNeo) showed a rate of 21% (with a variation of 12-28% between the different networks), showing a significant trend towards an increase in the use of medication.^(12)^

In Brazil, this issue has not been systematically evaluated, raising concerns about real estimates and potential long-term effects.

## OBJECTIVE

Therefore, we aimed to evaluate the rate of postnatal dexamethasone use in treatment of bronchopulmonary dysplasia and its trends in very low-birth-weight infants. Second, we evaluated the rate of use according to the gestational age (<32 and <28 weeks).

## METHODS

We performed a single-center retrospective cohort study using database analysis. All VLBW infants included in the database were eligible for inclusion. We included those born before 37 weeks of gestation and admitted between January 1, 2006, and December 31, 2022.

### Setting

This study was conducted in a tertiary university level III neonatal intensive care unit (NICU) with 30 beds and an annual admission rate of 650-750 newborn infants. Our NICU has an electronic database containing the clinical information of all VLBW infants born in 2006. Data were prospectively extracted from medical records and registered in a database whose clinical definitions followed the standards specified by the Brazilian Neonatal Research Network and the Vermont-Oxford Network.

### Variables

The main outcome was the postnatal use of dexamethasone, defined as parenteral and/or enteral use of the drug to treat BPD, regardless of the dose or duration of treatment. The clinical decision to use dexamethasone was based on the attending physician's judgment and reserved for infants who could not be weaned from mechanical ventilation after the first week of life, commonly associated with radiological changes compatible with BPD. Other indications for the use of dexamethasone, especially in the treatment of post-extubation laryngitis, were not evaluated in this study because they involved short cycles of use (three-five doses), and the adverse effects of the drug on neurodevelopment were associated with prolonged cycles and/or high cumulative doses. Dexamethasone protocols for the treatment of BPD have varied over the years, ranging from 42 days (high-dose regimen) to 9 days (recent low-dose schedule, total dose of 0.90 mg/kg).

To compare newborns with and without dexamethasone exposure, we analyzed the following maternal and obstetric descriptive variables: age, prenatal care, race (by self-declaration), presence of arterial hypertension, occurrence of diabetes mellitus, antenatal use of steroids (at any dose), obstetric hemorrhage, mode of delivery (vaginal or cesarean), and clinical chorioamnionitis.^(13)^

We compared descriptive neonatal variables: birth weight, gestational age (preferentially estimated by last menstrual period, early ultrasound estimative, or New Ballard score), sex, 1- and 5-minutes Apgar scores, resuscitation at delivery room (need for application of positive pressure ventilation by mask and/or tracheal tube), tracheal intubation at the delivery room, respiratory distress syndrome (respiratory symptoms and X-ray showing compatible to disease-reticulogranular infiltrate, with or without low lung volumes and air bronchogram), surfactant use, need for mechanical ventilation, noninvasive respiratory support, duration of use of oxygen and mechanical ventilation (days), oxygen use at discharge. We defined BPD as the requirement for supplemental oxygen (O^2^, >21% oxygen) at 36 weeks PMA, regardless of the respiratory support administered. For infants discharged, transferred, or those who died before 36 weeks PMA, the variable was classified as not applicable.

### Data analysis

We discuss the results for the entire cohort of VLBW infants and two gestational age subgroups: infants born at <32 and <28 weeks of gestation. Categorical variables were assessed using frequencies (percentages) and compared using the χ^2^ test or Fisher's exact test. Continuous variables were expressed as median and interquartile range (IQR) owing to the lack of normality of the data and were evaluated using the Mann-Whitney test. The annual rate of dexamethasone use was expressed as a percentage, considering the total number of VLBW infants admitted in the cited year, and was assessed using the Cochran-Armitage test. Statistical significance was set at p<0.05 was considered significant. Statistical analysis was conducted using SAS^®^ software (Version 9.4, SAS Institute, North Carolina, USA).

### Ethics

This study was approved by the local research ethics committee of *Universidade Estadual de Campinas* (CAAE: 83200017.0.0000.5404; #6.135.392). The requirement for informed consent was waived due to the retrospective nature of the study.

## RESULTS

A total of 1,691 VLBW infants were admitted to the NICU during the 17-year study period. The median birth weight was 1100g (IQR 850-1300), and the median gestational age was 29 weeks (IQR, 27-31 weeks). Finally, 1002 (59.3%) VLBW infants were discharged, and 428 (25.3%) were transferred to a secondary hospital, with a mortality rate of 15.4%.

A total of 163 infants (9.6%) were exposed to dexamethasone, with the annual variation ranging from 3.7% to 9.2%. Most infants exposed to dexamethasone remained dependent on supplemental oxygen at 36 weeks PMA (85%) (Table 1).

**Table 1:** Rate of use of dexamethasone in very low-birth-weight infants according to the diagnosis of bronchopulmonary dysplasia, using the time criteria (36 weeks postmenstrual age)

		Dexamethasone use	
		Yes n=163	No n=1,528
Oxygen use at 36 weeks postmenstrual age			
	Yes	139 (85.3)	236 (15.4)
	No	12 (7.4)	754 (49.3)
	Not applicable	12 (7.4)	538 (35.2)

Variables are expressed in absolute and relative frequency.

Infants exposed to dexamethasone differed significantly from those in the non-exposed group in terms of maternal and neonatal characteristics (Table 2). They were born less frequently by cesarean delivery (p<0.001) and were more likely to be exposed to clinical chorioamnionitis (p=0.005). They had a lower birth weight (p<0.001) and were more likely to be immature (p<0.001). In the delivery room, they had higher rates of Apgar scores <7 at 1 and 5 minutes (p<0.001) and a higher rate of resuscitation and tracheal intubation in the delivery room (p<0.001). In the NICU, patients presented with a higher frequency of respiratory distress syndrome (p<0.001), a higher rate of surfactant use (p<0.001), and a higher frequency of persistent ductus arteriosus and surgical closure (p<0.001). The utilization and median use of oxygen (p<0.001) and mechanical ventilation (p<0.001) were significantly higher in exposed infants. The duration of oxygen use, mechanical ventilation, and oxygen requirement at discharge (p<0.001) were more frequent in infants exposed to dexamethasone.

**Table 2:** Demographic characteristics of the cohort of very low-birth-weight infants

		Dexamethasone Yes (163)	Dexamethasone No (1,528)	p value*
Maternal variables				
	Age, years	28 (22-33)	28 (22-33)	0.434
	Prenatal care	152 (93.2)	1399 (91.6)	0.456
	White	87 (53.4)	801 (52.4)	0.817
	Arterial hypertension	60 (36.8)	607 (39.8)	0.454
	Diabetes mellitus	15 (9.2)	169 (11.1)	0.463
	Antenatal steroids	132 (80.1)	1186 (77.6)	0.325
	Cesarean delivery	95 (58.3)	1168 (76.4)	<0.001
	Peripartum hemorrhage	19 (11.7)	714 (7.5)	0.059
	Clinical chorioamionitis	18 (11.0)	85 (5.6)	0.005
Neonatal variables				
	Birth weight, gram	765 (640-920)	1134 (890-1320)	<0.001
	Gestational age, weeks	26 (25-28)	29 (28-31)	<0.001
	Apgar 1^st^ minute <7	42 (26.1)	194 (12.8)	<0.001
	Apgar 5^th^ minute <7	111 (68.9)	673 (44.9)	<0.001
	Resuscitation at the delivery room	99 (60.7)	593 (38.8)	<0.001
	Tracheal intubation	74 (45,9)	381 (25.7)	<0.001
	Respiratory distress syndrome	138 (84.7)	737 (48.9)	<0.001
	Surfactant	131 (80.4)	659 (43.1)	<0.001
	Mechanical ventilation	158 (96.9)	832 (54.7)	<0.001
	Oxygen use, days	91 (69-113)	6 (1-31)	<0.001
	Mechanical ventilation, days	34 (20-51)	1 (0-5)	<0.001
	Oxygen at discharge#	39 (34.2)	61 (6.9)	<0.001
	Persistence of ductus arteriosus	128 (78.5)	407 (26.6)	<0.001
	Surgical closure ductus arteriosus	54 (33.1)	55 (3.6)	<0.001
	Congenital malformation	8 (5.1)	85 (5.9)	0.689

* Categorial variables are expressed as absolute and relative frequencies and compared using the χ^2^ test or Fischer exact test. Continuous variables are expressed as median and interquartile range intervals and compared using the Mann-Whitney Test;

# n total = 1,002 - n dexamethasone 114.

**Figure 1:**
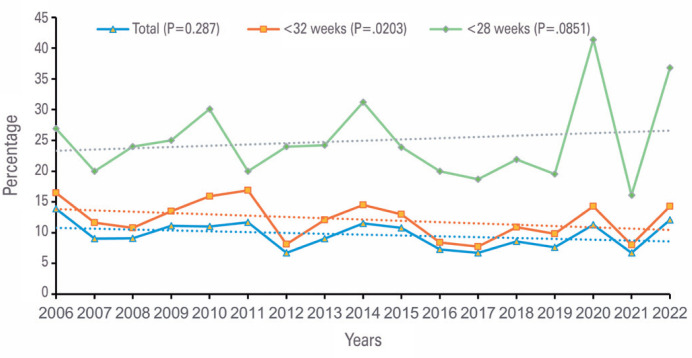
Trends for postnatal use of dexamethasone in very low-birth-weight infants (total sample) and in those younger than 32 and 28 weeks of gestation

The annual distribution of dexamethasone use varied from 6.7% to 13.9%, and there was no statistical difference between the years (p=0.287). For infants born at <32 weeks of gestation, the mean rate was 12.0%, and the annual rates varied between 7.7% and 16.9%, with no statistical difference (p=0.203). For more immature infants (<28 weeks of gestation), the mean rate was 24.6%, and there was no significant difference among the years (16.1%-41.4%, p=0.851) (Figure 1).

**Figure 2:**
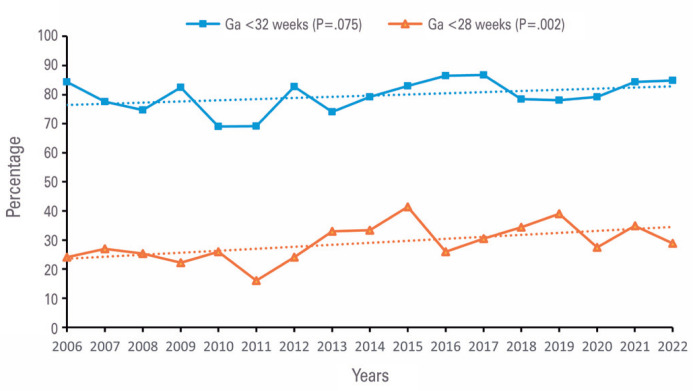
Trends in the incidence of births of infants younger than 32 and 28 weeks of gestation

We evaluated birth trends in infants younger than 32 and 28 weeks of gestation, as shown in figure 2. For infants younger than 32 weeks, the annual mean rate was 79.6% (varying from 69.0% to 86.5%) and was stable over time (p=0.075). The annual mean rate for infants younger than 28 weeks of gestation was 29.0 (annual variation 16.0-41.4%) and showed a statistically significant increasing trend over time (p=0.002).

**Figure 3:**
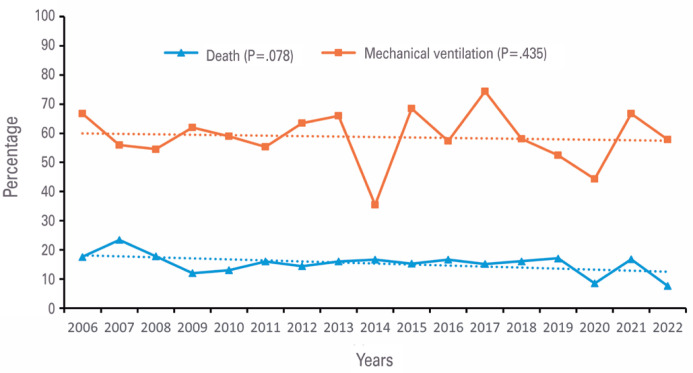
Trends in death rate and mechanical ventilation in very low birth weight infants from 2006 to 2022

As shown in figure 3, considering the trends in in-hospital death rate (p=0.078) and mechanical ventilation use (p=0.435), we did not observe any statistically significant differences during this period.

## DISCUSSION

In this single-center retrospective analysis, we observed that the postnatal use rate of dexamethasone in VLBW infants was low; one in ten infants received the drug during their stay in the NICU. As expected, in more immature infants, this rate was more relevant, reaching one in four of those born at <28 weeks of gestation; however, even in this subgroup, there was stability in the rate of use.

The dexamethasone rate observed in infants born at <32 weeks of gestation was more than double that observed in a multicenter evaluation by Yao et al. in 62,019 infants in England and Wales between 2012 and 2019 (5.3%).^(10)^ However, the postnatal use of any drug over the years (4.5-6.6%, p=0.04), reached a similar rate of 27 weeks, whereas the postnatal use of any steroid for BPD, most commonly dexamethasone, reached a similar rate of 26%.

Kwok et al.^(14)^ performed another extensive evaluation of postnatal steroid use in England and Wales, evaluating 83,463 preterm infants born at <32 weeks’ gestation from 2010 to 2020. The global rate of dexamethasone use was less than half that of our results (5%); however, they demonstrated an increase from 4% in 2010 to 7% in 2020 (p<0.001). In premature infants born before 28 weeks of gestation, the mean rate was higher, presenting a significant increase from 11% in 2010 to 18% in 2020 (p<0.001).

In another multicenter evaluation of infants born at <32 weeks of gestation in France by Iacobelli et al., the rate of exposure to postnatal steroids was higher than in our study (21.6%). Nevertheless, following local recommendations, betamethasone is the most commonly used steroid in that population. Dexamethasone use occurred in only six infants among more than 13,000 preterm newborns.^(11)^

In a recent Korean cohort of 11,261 very-low-birth-weight infants born between January 2013 and December 2020, with a gestational age of 23-31 weeks, the prevalence of dexamethasone use was higher at 20.5% (annual variation 17.7-22.3%).^(15)^

Parikh et al. analyzed the postnatal use of steroids in seven international networks participating in iNeo: Canada, Finland, Israel, Japan, Spain, Sweden, and Switzerland. A global evaluation analyzed 47,401 neonates born between 24 and 28 weeks of gestation. This mean rate is comparable to our result (21%), varying from 12% to 28% across national networks. They significantly increased over time (from 18% in 2010 to 26% in 2018; p<0.01).^(12)^

Only one report on this subject was published in a low- and middle-income country. In a multicenter cohort of 16 South American hospitals, namely NeoCosur Red, Tapia et al. analyzed 1,825 very low-birth-weight infants whose mean birth weight and gestational age were 1085±279g and 29±3 weeks, respectively. The postnatal steroid administration to infants with BPD decreased during the study period, from 46% in 2000 to 16% in 2003 (p<0.05). However, recent publications on the NeoCosur Network have not addressed this issue.^(16)^

These contemporary articles highlight concerns regarding the long-term effects of dexamethasone exposure, specifically related to its association with cerebral palsy. As described by Cochrane metanalysis, early (less than 7 days of life) exposure to steroids increased the risk of cerebral palsy (risk ratio [RR] 1.43, 95% confidence interval [CI] 1.07–1.92; 13 studies, 1,973 infants; high-certainty evidence), as did dexamethasone (RR= 1.77, 95%CI= 1.21–2.58; 7 studies, 921 infants; high-certainty evidence). After the first week of life, the use of systemic corticosteroids reduced both BPD and mortality without any evidence of long-term neurological harm. However, the authors highlighted that the methodological quality of the studies regarding long-term outcomes was limited, and none of the studies detected increased rates of adverse long-term neurodevelopmental outcomes. Therefore, clinicians should analyze the risks and benefits of a late and short course of dexamethasone to promote difficult tracheal extubation after the second week of life.^(6,7,17)^ Accordingly, the restricted or rational use of dexamethasone falls within the context of neuroprotective strategies, as defined by Carteaux et al.^(18)^ and is a criterion that can be used in care quality improvement programs.^(19)^

Bronchopulmonary dysplasia has various clinical definitions because of its multifactorial nature and variable clinical presentation, making it difficult to compare the disease rate and severity observed in different studies.^(20)^ In this evaluation, the BPD rate was defined by a temporal limit (36 weeks PMA); however, it is important to highlight that the decision on whether to administer dexamethasone was a clinical decision made before this limit, as the drug is administered in the second or third week of life, generally before 36 weeks PMA, in an attempt to promote tracheal extubation. Moreover, 85% of the children exposed to dexamethasone required supplemental oxygen at 36 weeks’ PMA and had a significantly higher rate of home discharge with supplemental oxygen, demonstrating the severity of the disease.

A limitation of our study is its external validity owing to its retrospective nature. Therefore, it is not possible to compare our drug use rates with those reported in other Brazilian hospitals. However, this is the first report in Brazil on the rate of dexamethasone use in a significant number of very low-birth-weight infants. Another limitation was the impossibility of assessing the timing of medication use owing to the retrospective nature of the database evaluation.

Regarding the extent of the dexamethasone cycles, there was a significant modification in the local routine in June 2013, limiting it to a single 9-day course, totaling 0.9mg/kg (0.15mg/kg 3 days- 0.10mg/kg 3 days-0.05mg/kg 3 days), applied to infants with severe disease or those whose extubation was not possible. The neonatal score was not used in the BPD risk unit to indicate the use of dexamethasone. A clinical evaluation of severity was performed.

## CONCLUSION

In conclusion, approximately one in every 10 preterm very low-birth-weight infants and one in every four those younger than 28 weeks of gestation received dexamethasone, with a trend towards stable use over the period, despite the significant trend towards an increase in extreme preterm newborns.
